# Identification and validation of a prognostic signature related to hypoxic tumor microenvironment in cervical cancer

**DOI:** 10.1371/journal.pone.0269462

**Published:** 2022-06-03

**Authors:** Chenyu Nie, Haixia Qin, Li Zhang

**Affiliations:** 1 School of Public Health, Xinxiang Medical University, Xinxiang, Henan, China; 2 Department of Gynecology, The First Affiliated Hospital of Xinxiang Medical University, Weihui, Henan, China; Massachusetts Eye and Ear Infirmary, Harvard Medical School, UNITED STATES

## Abstract

**Background:**

Hypoxia is a common microenvironment condition in most malignant tumors and has been shown to be associated with adverse outcomes of cervical cancer patients. In this study, we investigated the effects of hypoxia-related genes on tumor progress to characterize the tumor hypoxic microenvironment.

**Methods:**

We retrieved a set of hypoxia-related genes from the Molecular Signatures Database and evaluated their prognostic value for cervical cancer. A hypoxia-based prognostic signature for cervical cancer was then developed and validated using tumor samples from two independent cohorts (TCGA-CESC and CGCI-HTMCP-CC cohorts). Finally, we validated the hypoxia prediction of ccHPS score in eight human cervical cancer cell lines treated with the hypoxic and normoxic conditions, and 286 tumor samples with hypoxic category (more or less) from Gene Expression Omnibus (GEO) database with accession GSE72723.

**Results:**

A risk signature model containing nine hypoxia-related genes was developed and validated in cervical cancer. Further analysis showed that this risk model could be an independent prognosis factor of cervical cancer, which reflects the condition of the hypoxic tumor microenvironment and its remodeling of cell metabolism and tumor immunity. Furthermore, a nomogram integrating the novel risk model and lymphovascular invasion status was developed, accurately predicting the 1-, 3- and 5-year prognosis with AUC values of 0.928, 0.916 and 0.831, respectively. These findings provided a better understanding of the hypoxic tumor microenvironment in cervical cancer and insights into potential new therapeutic strategies in improving cancer therapy.

## Introduction

Cervical cancer (CC) is the fourth leading cause of cancer incidence and mortality among women worldwide [1]. Despite the increased use of cervical cancer screening and HPV vaccines, and the improvement of diagnostic and treatment techniques, cervical cancer is still a public health problem [1–3]. Especially in the low-resources countries, such as countries in Africa, cervical cancer remained as the leading cause of cancer-related death because of late diagnosis at invasion stages [1, 4]. Thus, it is necessary to explore and identify the molecular mechanisms of carcinogenesis in cervical cancer, which may lead to new therapeutic strategies, and thereby improve the survival of patients with cervical cancer.

Hypoxia is a biological condition in which adequate oxygen supply is deprived at the tissue level, a common microenvironment feature in most malignant tumors [5, 6]. Tumor hypoxia results from rapid proliferation, altered tumor cell metabolism, and abnormal surrounding vasculature in the tumor microenvironment [7, 8]. Hypoxia causes a series of changes in biological functions [9, 10], leading to more therapeutically resistant tumor cells with increased aggressive progression [5, 7].

Many reports have confirmed that hypoxia is a prognosis factor associated with adverse outcomes in cervical cancer [11–13]. Several hypoxia-related genes have been identified as robust biomarkers in predicting overall survival (OS) of patients with cervical cancer, such as hypoxia-inducible factor-1 alpha (HIF-1α) [14–16]. However, previous studies mainly focused on investigating cancer prognosis with one or a limited number of hypoxia-related genes [17–19]. As hypoxia is a complex microenvironment that induces a series of biological changes in cell metabolism, proliferation, and apoptosis [5, 6], integrated analyses of hypoxia-related genes will provide a more comprehensive understanding of hypoxia-induced biological changes and their effects in cancer progression, ultimately improving cancer therapy. A recent publication from Yang et al. [20] constructed a 5-gene prognostic signature in cervical cancer based on the molecular subtype clustering using hypoxia hallmark genes, and the five genes were further experimentally verified to be potential prognostic targets respectively. However, the relationship between this signature and the hypoxic tumor condition was not verified, and this signature did not give us more insights about the biological changes in tumor microenvironment under hypoxia.

In this study, we investigated the effects of hypoxia-related genes on tumor progress to construct a hypoxia-related gene signature, which could be an independent prognostic factor. Moreover, we proved that this signature is associated with tumor hypoxia level, high risk score reflects the relatively severe tumor hypoxia. Importantly, we investigated the characteristics of the tumor hypoxic microenvironment between high- and low-risk patients, including their chemotherapy drug sensitivity, and biological changes of the tumor microenvironment (TME) in cell metabolism and tumor immunity. The results will provide a better understanding of the hypoxic tumor microenvironment in cervical cancer and insights into potential new therapeutic strategies in improving cancer therapy. In details, we retrieved a set of hypoxia-related genes in the Molecular Signatures Database, investigated their prognostic value in cervical cancer, and finally developed and validated a Hypoxia-related Prognostic Signature for cervical cancer (ccHPS) using tumor samples in TCGA-CESC and CGCI-HTMCP-CC cohorts. Significantly, the ccHPS risk model in this study has a robust performance in predicting OS for patients with cervical cancer, and could serve as an independent prognostic factor. Combining with eight human cervical cancer cell lines treated with the hypoxic and normoxic condition, and 286 tumor samples with hypoxic category (more or less) from GSE72723, we verified that the ccHPS model could reflect the condition of hypoxic tumor microenvironment. Furthermore, we investigated the biological changes of the tumor microenvironment (TME) in cell metabolism and tumor immunity by comparing the high-risk and low-risk patients classified by the ccHPS risk model, and showed that the ccHPS model might act as an indicator for TME remodeling.

## Materials and methods

### Data collection

The hypoxia-related gene (HRG) set was downloaded from hallmark gene sets in the Molecular Signatures Database (MSigDB) [21], which includes 200 genes in response to hypoxia.

RNA sequencing (RNA-Seq) data and corresponding clinical metadata of tumor samples from the cohort of TCGA cervical squamous cell carcinoma and endocervical adenocarcinoma (TCGA-CESC) were retrieved from the UCSC Xena Browser (https://xenabrowser.net/datapages/). The clinical metadata includes Age, Grade, TNM stage classification, FIGO stages, lymphovascular invasion (LVI) and radiation therapy. RNA-Seq data and survival information of samples from an independent cohort of CGCI HIV+ Tumor Molecular Characterization Project (CGCI-HTMCP-CC) were obtained from TCGA using the R package ‘TCGAbiolinks’. For the CGCI-HTMCP-CC samples, if multiple samples from the same cervical cancer cases, we randomly selected one of them (S1 Table). Both TCGA-CESC and CGCI-HTMCP-CC RNA-Seq data were normalized as fragments per kilobase of transcript per million mapped reads (FPKM) and then log2-transformed. The Ensembl gene identifiers were converted into gene symbols according to the gene mapping file (S2 Table), which was extracted from the gene annotation file (http://ftp.ebi.ac.uk/pub/databases/gencode/Gencode_human/release_22/gencode.v22.annotation.gtf.gz) in the Human GENCODE database (version 22) [22]. If a gene symbol mapped to multiple Ensembl gene identifiers, the median expression value was selected.

The expression array data of 286 tumor samples and 16 cell line samples in Gene Expression Omnibus (GEO) database with accession GSE72723 [18, 19] were downloaded using R library ‘GEOquery’. Of the above 302 expression array samples, 150 samples and 4 cell line samples with Illumina WG-6 were mapped into gene symbols according to the annotation for the GPL6884 platform. At the same time, 136 tumor samples and 12 cell line samples with Illumina HT-12 were mapped according to the GPL10558 platform. When multiple probes corresponding to the same gene, the median expression value was selected.

### Development of the hypoxia-related gene risk model

In the process of model development, we selected the 289 patients that included both clinical data and gene expression profile data in TCGA-CESC. The univariate Cox analysis was performed in the TCGA-CESC cohort (n = 289) to select significant HRGs with prognostic value in cervical cancer, adopted using the ‘survival’ package in R with *P*-value < 0.01 as the significant threshold. The 289 TCGA-CESC patients were randomly assigned into a training cohort (n = 203) and a test cohort (n = 86) at a 7:3 ratio using the ‘caret’ package (S1 Table). The training cohort was used to develop the risk model, whereas the test cohort was used as one of the two validation sets. Besides, 117 tumor samples with survival information from cohort CGCI-HTMCP-CC were used as additional validation cohort to qualify model performance. The clinical characteristics of patients in the training and two test cohorts are summarized in S3 Table.

Firstly, the least absolute shrinkage and selection operator (LASSO) method was used to fit the most informative and parsimonious Cox regression model using R software package ‘glmnet’ in the TCGA-training cohort, and establish the best combination of the HRGs. The Penalty parameter (λ) for the model was determined from tenfold cross-validation using the minimum criteria, this minimum value of λ is corresponding to the lowest partial likelihood deviance. Subsequently, Hypoxia-related Prognostic Signature for cervical cancer (ccHPS) was developed to predict the risk score by combining the coefficients of HRGs in the LASSO model and the expression abundance of HRGs in each tumor sample. The formula was as follow:

$$ccHPS=\sum_{i=1}^{n}\left({Gene\_expression}_{i}\times {Coefficinet}_{i}\right)$$

Finally, Principal Component Analysis (PCA) was performed base on the expression of genes in the ccHPS model. The time-dependent receiver operating characteristic (ROC) curves and Kaplan-Meier (K-M) survival analysis were conducted to assess the prognostic accuracy of the ccHPS risk score using ‘survivalROC’ and ‘survival’ package in TCGA-training cohort (Training set), TCGA-test cohort (Validation set-1) and an independent dataset from CGCI-HTMCP-CC cohort (Validation set-2).

### Evaluation of immune status between high-risk and low-risk groups

The immune score and tumor purity for each patient sample were calculated using the ESTIMATE (Estimation of STromal and Immune cells in Malignant Tumors using Expression data) algorithm with ‘estimate’ package [23], which uses gene expression data to infer the fraction of stromal and immune cells in tumor samples. Estimation of immune cell infiltration fractions was conducted using the CIBERSORT (Cell type Identification By Estimating Relative Subsets Of RNA Transcripts) method [24], which characterizes the immune cell composition (22 immune cell types) of a tumor biopsy from their gene expression profiles. The significance values were determined based on 100 permutations. Samples with *P*-value < 0.05 in CIBERSORT were selected for further analysis. Microenvironment Cell Populations-counter (MCP-counter) method [25] was used to quantify the abundance of immune cells and stromal cells (T cells, CD8^+^ T cells, Cytotoxic lymphocytes, B lineage, NK cells, Monocytic lineage, Myeloid dendritic cells, Neutrophils and Endothelial cells) in tumors of each patient from gene expression profiles. We compared the differences in ESTIMATE, CIBERSORT and MCP-counter results between high- and low-risk patients by Student’s t-test.

### Gene set enrichment analysis

All genes in TCGA-CESC dataset were first processed by log2 transformation and then ranked concerning their differential expression between high- and low-risk patients using ‘limma’ package [26]. Gene set enrichment analysis (GSEA) was conducted between high- and low-risk samples to investigate the functional divergence in two groups, using the R package ‘clusterProfiler’ with 20,000 permutations [27–29]. Gene sets derived from Gene Ontology (biological process), KEGG pathways and HALLMARK pathways were collected from MSigDB. The Benjamini-Hochberg (BH) method was used to control the false discovery rate [30]. Adjusted *P*-value < 0.05 and the NES were used to determine the enrichment of a pathway between two groups.

### Chemosensitivity prediction

The chemotherapeutic sensitivity for each tumor sample was estimated based on the pharmacogenomics data in Genomics of Drug Sensitivity in Cancer database (GDSC; https://www.cancerrxgene.org/) [31]. The half-maximal inhibitory concentration (IC50) of each treated specific chemotherapy drug in each tumor sample was estimated using the R package ‘pRRophetic’ [32, 33].

### Nomogram construction and evaluation

A nomogram incorporating the risk scores and clinical characteristics of patients was constructed using the ‘rms’ package. A concordance index (C-index) was calculated to assess the discrimination of the nomogram via a bootstrap method with 1000 resamples. The time-dependent receiver operating characteristic (ROC) curves were performed using the ‘survivalROC’ package. Decision Curve Analysis (DCA) was performed using ‘rmda’ package.

## Results

### Identification of prognostic hypoxia-related genes

**Fig 1:**
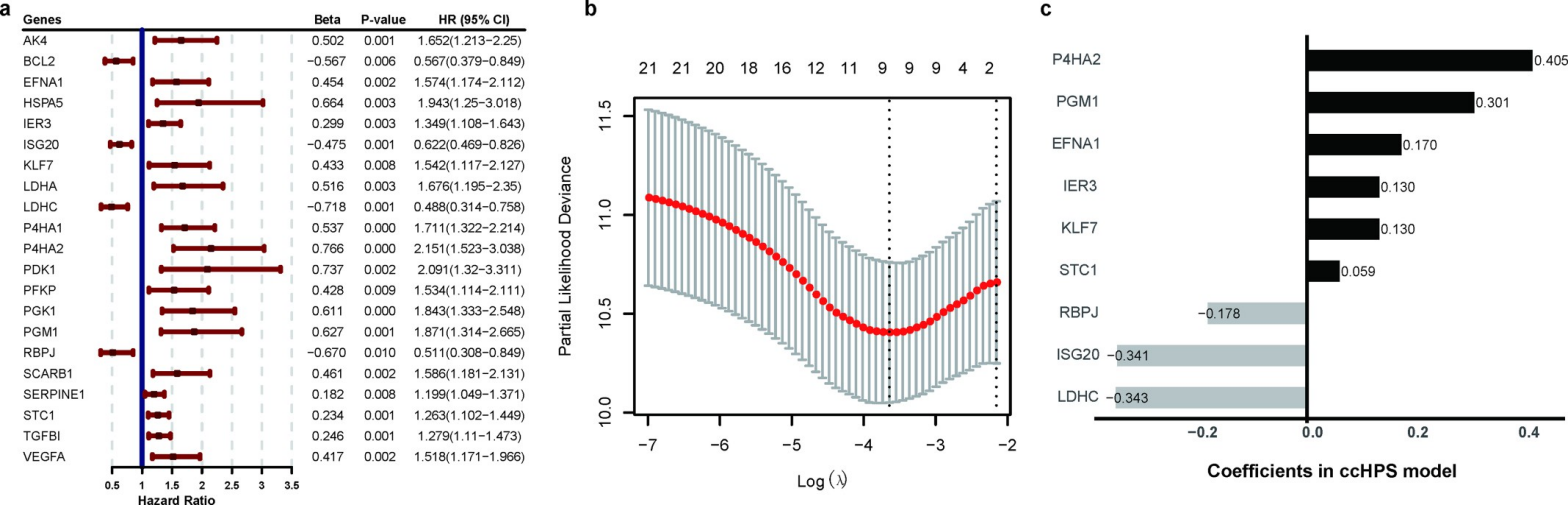
Identification of the hypoxia-related genes to develop a risk model. (a) Forest plot showing the significant HRGs in Univariate Cox regression analysis with P < 0.01 using TCGA-CESC entire cohort. (b) Partial likelihood deviance for tuning the parameter selection in the LASSO regression model using training cohort. The red dotted line is the cross-validation curve, and the error bars are the upper and lower standard deviation curves along the λ sequence. The two dotted vertical lines represent the optimal values by minimum criteria (left) and 1-se criteria (right). The minimum criteria was selected in this study. (c) The coefficients of each gene in the ccHPS model.

Of 200 hypoxia-related genes (HRGs) from MSigDB, 190 were covered in the TCGA-CESC dataset (S4 Table). To further investigate the prognostic-related HRGs in cervical cancer, we performed survival analysis for each HRG by comparing patients’ overall survival (OS) status in low- and high-expression groups based on their median expression. As a result, 21 genes were found significantly related to OS (*P* < 0.01) and were considered as prognostic-related genes for further analysis (Fig 1A and S4 Table).

### Construction of hypoxia-related prognostic risk model

Based on the above 21 prognostic HRGs, we conducted LASSO Cox regression analysis to develop a Hypoxia-related Prognostic Signature for cervical cancer (ccHPS) using the training cohort. As a result, a prognostic risk model with 9 HRGs (EFNA1, IER3, ISG20, KLF7, LDHC, P4HA2, PGM1, RBPJ and STC1) was identified according to the optimal value of lambda (log(λ) = -3.34; Fig 1B). The coefficients of each gene in the ccHPS model were shown in Fig 1C. There were six genes positively associated with risks and three genes negatively associated.

**Fig 2:**
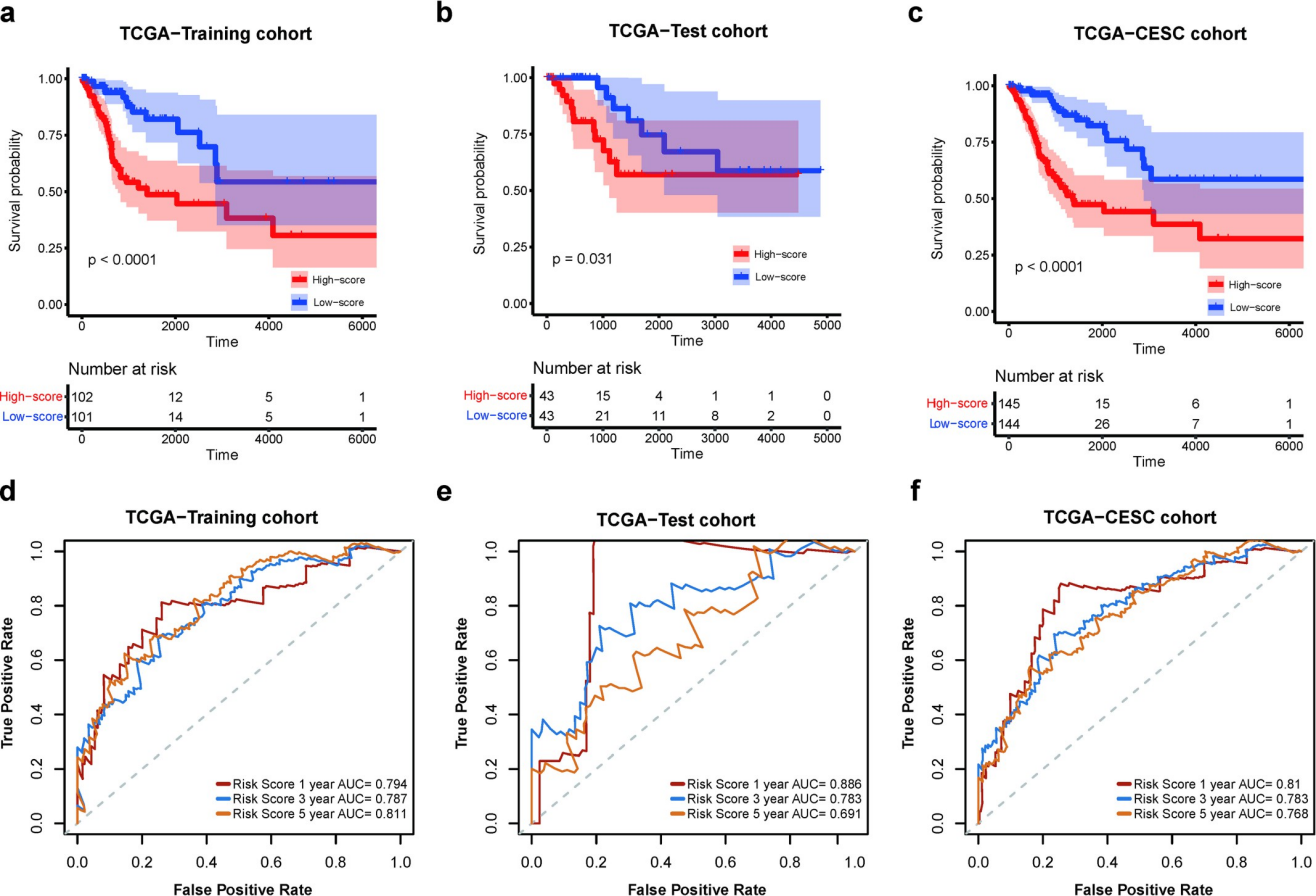
Evaluation of the prognostic prediction ability of ccHPS risk model in both training and validation sets in TCGA-CESC cohort. (a-c) Kaplan-Meier curves for the OS of patients in the TCGA-training cohort (a), TCGA-test cohort (b), and TCGA-CESC entire cohort (c) between the high-risk and low-risk groups that were separately divided by ccHPS risk model. (d-f) Time-dependent ROC curves for the ccHPS risk model at 1-, 3- and 5-years survival time in the TCGA-training cohort (d), TCGA-test cohort (e), and TCGA-CESC entire cohort (f), respectively.

To evaluate the performance of the ccHPS risk model, the risk scores for each patient in the training cohort were then calculated according to the gene expression of 9 signature genes in each patient and gene coefficients in the risk model. According to the median risk score, patients were divided into two groups (high- and low-risk groups). PCA analysis showed that patients in the two risk groups were distributed in two directions (S1A Fig). K-M survival analysis indicated that high-risk patients had a significantly shorter survival time than low-risk patients (*P* < 0.01, Fig 2A). Moreover, the time-dependent ROC curve was performed to assess the performance of risk score in predicting OS (Fig 2D). The result showed that the ccHPS model could predict the 1-, 3- and 5-year prognosis with AUC values of 0.79, 0.79 and 0.81, respectively. These findings suggested that ccHPS can predict OS in cervical cancer, and a high ccHPS score was associated with a poor clinical outcome.

### Validation of the stable performance and prognostic value of ccHPS

The TCGA-test cohort (n = 86), the TCGA-entire set (n = 289), and an additional independent CGCI-HTMCP-CC cohort (n = 117) were further used to assess the robustness of the ccHPS model.

Of the TCGA-test cohort and the TCGA-entire set, the risk score of each patient was calculated using the same ccHPS formula as above, and patients were subsequently divided into high- and low-risk groups according to their median scores. PCA analysis confirmed that patients in high- and low-risk groups were clustered in discrete directions in both TCGA-test and the entire set (S1b and S1c Fig). Consistent with the training dataset, the high-risk group significantly had a shorter predicted survival time than low-risk groups (Fig 2B and 2C). Moreover, the time-dependent ROC curve was conducted in both sets. In the TCGA-test set, the AUC values of 1-, 3- and 5-years are 0.89, 0.78 and 0.69, respectively (Fig 2E). In the TCGA-entire set, the AUC values of 1-, 3- and 5-years are 0.81, 0.78 and 0.77, respectively (Fig 2F). The results suggested that the current prognostic model is relatively stable, especially in year 3. Besides, we found AUC is decreased in test sets during follow-up, AUC is highest in year 1 and lowest in year 5. As both the disease status of an individual and biomarker values may change during follow-up, therefore, the most recent marker value may be best related to the current disease status of an individual [34, 35].

**Fig 3:**
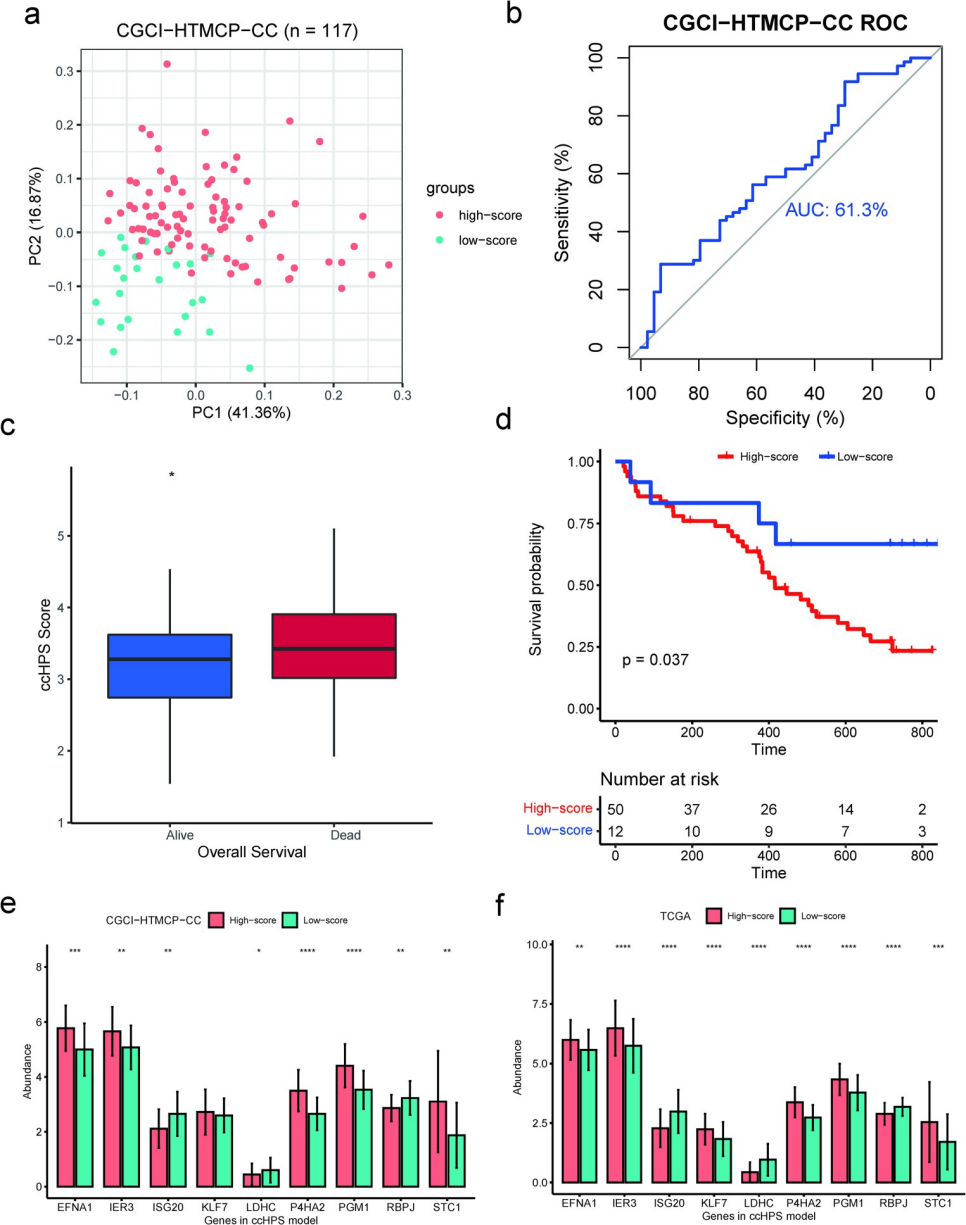
Validation of the prognostic value of ccHPS model using an independent CGCI-HTMCP-CC cohort. (a) Principal component analysis of the gene expression profile in high-risk and low-risk patients in CGCI-HTMCP-CC cohort. (b) ROC curves for the ccHPS risk model in the CGCI-HTMCP-CC cohort. (c) ccHPS score was significantly higher in patients with Dead OS status than that with Alive in the CGCI-HTMCP-CC cohort. (d) Kaplan-Meier curves for the OS of patients in the CGCI-HTMCP-CC cohort. (e) Expression level comparison of nine genes in ccHPS model using CGCI-HTMCP-CC cohort. The mean and standard deviation of gene expression levels were shown in the bar plot. (f) Expression level comparison of nine genes in ccHPS model using TCGA-CESC cohort. Statistical comparison in (c), (e), and (f) was performed using the Wilcoxon test. **** *P* ≤ 0.0001, *** *P* ≤ 0.001, ** *P* ≤ 0.01 and * *P* ≤ 0.05.

Furthermore, the prognostic value of the ccHPS model was validated using an additional dataset from the CGCI-HTMCP-CC cohort, which includes 117 cases with both RNA-Seq data and their overall survival. Consistently, PCA analysis confirmed that patients in high- and low-risk groups were clustered in discrete directions in the CGCI-HTMCP-CC cohort (Fig 3A). The AUC value in the CGCI-HTMCP-CC cohort is 0.61 (Fig 3B). The ccHPS score in the Dead (73 cases) was significantly higher than that in Alive (44 cases) (Fig 3C; *P* = 0.02). According to the quartile values (Q75 and Q25) in the TCGA-training cohort, we conducted the K-M survival analysis between high (higher than Q75) and low (lower than Q25) risk patients in CGCI-HTMCP-CC. Consistent with the TCGA cohort, the result showed that the high- and low-risk cases had statistical differences in survivals, and high-risk patients from CGCI-HTMCP-CC had poor clinical outcomes (Fig 3D). Besides, the distribution of the expression of the nine genes in the ccHPS model between high- and low-risk patients was investigated in both TCGA-CESC (Fig 3F) and CGCI-HTMCP-CC (Fig 3E) cohorts. All the nine genes had significant divergent expression levels between high- and low-risk groups in TCGA-CESC, and eight of nine genes in CGCI-HTMCP-CC were significantly divergent between two groups. The above results demonstrated the robustness and predictive ability of the ccHPS model.

Additionally, we investigated the stability of the ccHPS model across the clinical factors using the TCGA-CESC entire dataset. Each of the clinical factors was divided into two groups, and the included clinical factors are Age at diagnosis (> 50 and < = 50 years old), grade (G1-2 and G3-4), N stage (N0 and N1) and T stage (T1 and T2-4). Across all the clinical factors, the high-risk patients were significantly associated with a poor clinical outcome than that in the low-risk patients (*P* < 0.05; S2 Fig), suggesting a stable performance of the ccHPS model across the clinical factors.

### ccHPS reflects the hypoxic tumor microenvironment

**Fig 4:**
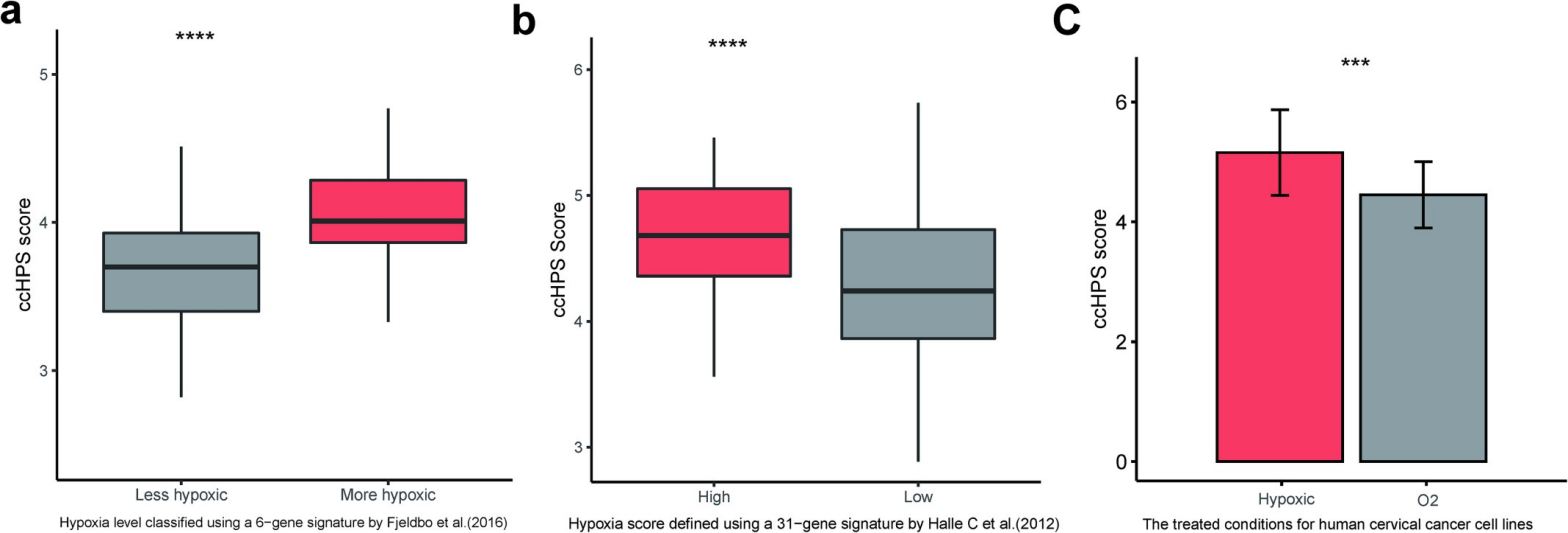
Validation of the tumor hypoxic microenvironment prediction ability of ccHPS model. (a) ccHPS score in more hypoxic tumors was higher than that in less hypoxic tumors, and the hypoxia level was classified using a 6-gene signature by Fjeldbo et al. [18]. (b) ccHPS score in tumors with high hypoxia score was higher than that in lower hypoxia levels, and the hypoxia score was classified using a 31-gene signature by Halle et al. [19]. (c) ccHPS score in human cervical cancer cell lines that were separately treated with hypoxic and normoxic conditions. The transcriptome data of eight cell lines were from Fjeldbo et al. [18], which include Hela, SW756, C-33, C-41, ME-180, HT-3, SiHa and CaSki, and each of them was treated with hypoxic and normoxic conditions respectively. Statistical comparison between the paired hypoxic and normoxic cells was performed using paired t-test. Statistical comparison between high- and low-score groups (in a and b) was performed using the Wilcoxon test. **** *P* ≤ 0.0001, *** *P* ≤ 0.001, ** *P* ≤ 0.01 and * *P* ≤ 0.05.

In addition to the prognostic value of the ccHPS model, we also investigated its reflection of the hypoxic TME by comparing it with two cervical cancer-specific hypoxia signatures, a 31-gene signature by Halle et al. [19] and its 6-gene reduced form by Fjeldbo et al. [18]. Comparing with Halle et al. [19], the ccHPS score in their defined high hypoxia tumors (77 samples) was significantly higher than that in low hypoxia tumors (73 samples) with *P*-value = 1.68e-06 (Fig 4A). For Fjeldbo et al. [18], the ccHPS score in the classified more hypoxic tumors (44 samples) was significantly higher than the less hypoxic tumors (92 samples) with *P*-value = 5.506e-07 (Fig 4B). Furthermore, we also validated the hypoxia prediction of ccHPS score in eight human cervical cancer cell lines (Fig 4C), each of them was separately treated with hypoxic (0.2% O_2_, 5% CO_2_) and normoxic (95% air, 5% CO_2_) conditions [18]. As shown in Fig 4C, the ccHPS score of hypoxic treated cells was significantly higher than the compared normoxic treated cells with *P*-value = 1.85e-04. The above results suggested the ability of the ccHPS model in predicting tumor hypoxic microenvironment.

### Function enrichment analysis

**Fig 5:**
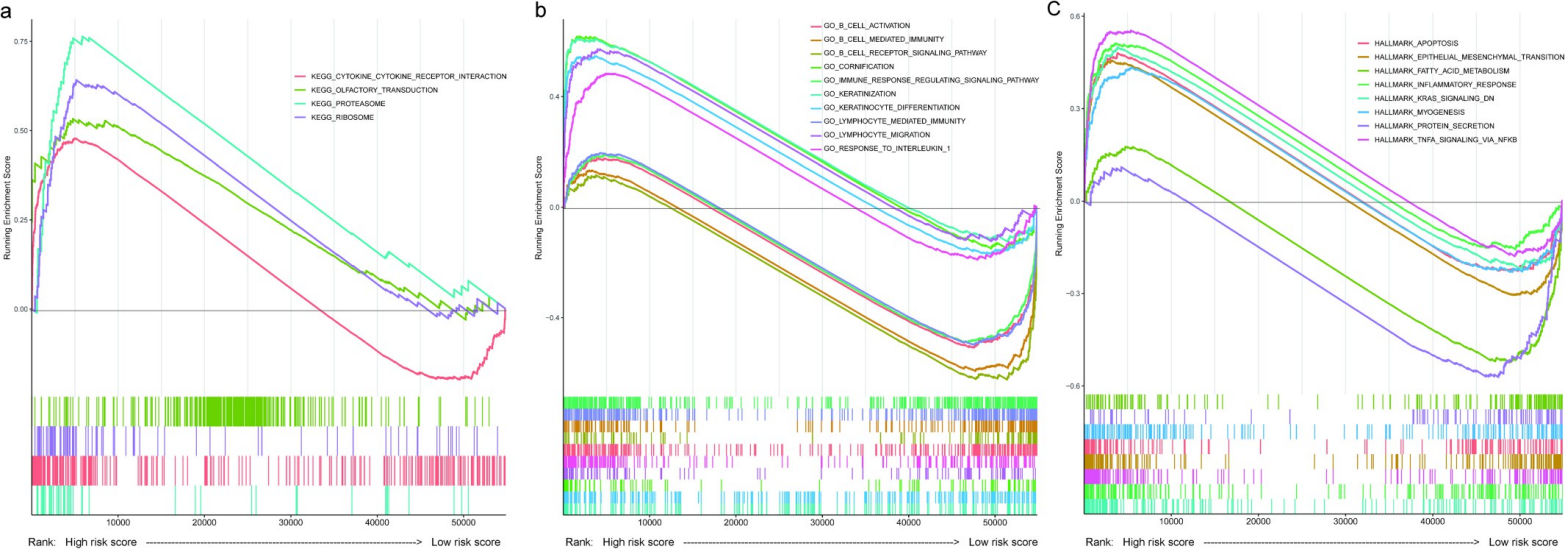
Functional enrichment analysis using GSEA for the high-risk and low-risk patients from the ccHPS risk model. (a) Enriched gene sets in KEGG pathways. Each line with different colors represents a specific gene set. Terms with adjust *P*-value < 0.05 were selected and considered to be enriched significantly. Genes ranked from left to right of the x-axis represent high-risk score to low-risk score. The four KEGG terms are enriched in high-risk patients. (b) Selected enriched gene sets in GO (biological process), which is part of GO (biological process) results in S5 Table. (c) Enriched gene sets in HALLMARK pathways. The detailed information about the enrichment scores and adjust *P*-values were listed in S5 Table.

We hypothesized that the cell metabolism in high- and low-risk patients is divergent, leading to different clinical outcomes in cervical cancer. To explore the functional divergence between high- and low-risk patients, we conducted Gene Set Enrichment Analysis (GSEA) using the Gene Ontology (biological process), KEGG pathways and HALLMARK gene sets that were derived from the MSigDB gene sets. At the GO biological process level, 47 pathways were enriched in low-risk patients and 17 in high-risk patients (S5 Table). At the KEGG pathway level, four pathways were enriched in high-risk patients (Fig 5A). For the HALLMARK gene signatures, six gene signatures were enriched in high-risk patients and two signatures in low-risk patients (Fig 5C).

As shown in Fig 5 and S5 Table, pathways in cervical cancer tumor progression and metastasis, such as epithelial-mesenchymal transition (EMT) [36], apoptosis and fatty acid metabolism [37], were enriched in high-risk patients. Besides, cytokine-cytokine receptor interaction pathway, ribosome, and three keratinization-related biological pathways, including keratinization, keratinocyte differentiation and cornification, and keratinization, were enriched in high-risk patients. Notably, all the above pathways were reported to be associated with cancer progression. For instance, keratinization was shown to be related to adverse outcomes in several cancers [38, 39]. Lappano et al. [40] reported that IL-1β (Interleukin-1 Beta), the critical component of the cytokine-cytokine receptor interaction pathway, contributes to the initiation and progression of breast cancer. Ribosomal dysfunction is related to tumor progression [41–43]. Further investigation of the enriched pathways in high-risk patients might give insights into the molecular mechanism underlying poor prognosis.

Interestingly, a series of immune-related pathways were enriched in low-risk patients, including B cell-mediated immunity, B cell receptor signaling pathway, immune response regulating signaling pathway, and B cell activation (Fig 5B and S5 Table).

### Alterations of tumor immune microenvironment between high- and low-risk patients

**Fig 6:**
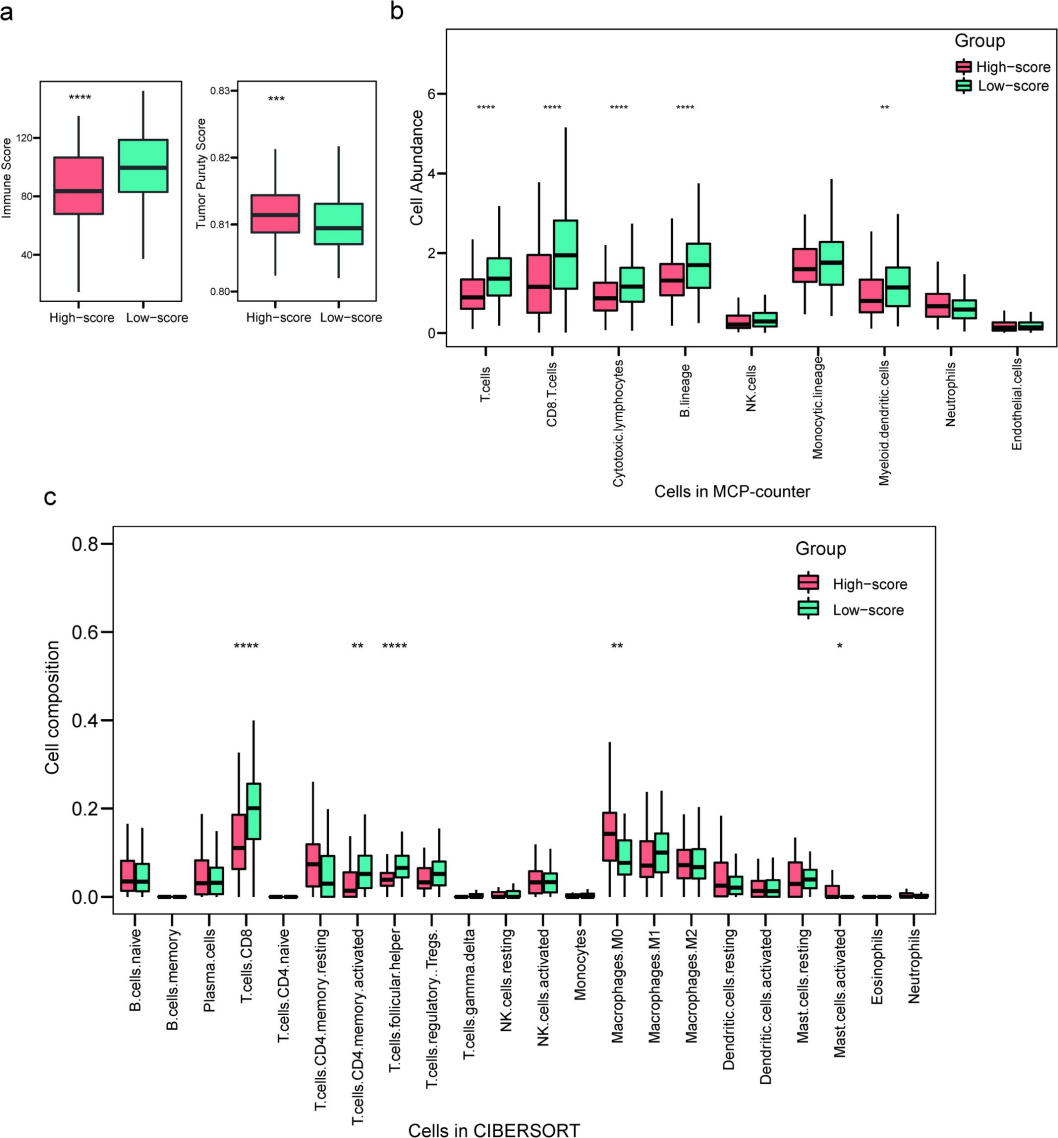
Comparison of the immune status between high-risk and low-risk patients from ccHPS risk model base on ESTIMATE, MCP-counter and CIBERSORT methods. (a) Boxplot shows the Immune Score distribution (left) and Tumor purity (right) from ESTIMATE. (b) Boxplot shows immune cell abundance in MCP-counter method (c) Boxplot shows the cell composition ratio differentiation of 21 types of immune cells in cervical cancer samples between high and low-risk patients using CIBERSORT. The statistical comparisons were conducted using Student’s t-test. **** *P* ≤ 0.0001, *** *P* ≤ 0.001, ** *P* ≤ 0.01 and * *P* ≤ 0.05.

To further explore the relationship between ccHPS risk score and immune status in patients, we evaluated the immune score between the high- and low-risk patients using ESTIMATE (Fig 6A). A significantly higher immune score was observed in low-risk patients than that in high-risk patients (*P* < 0.0001; Fig 6A), consistent with the GSEA result that a series of immune-related pathways were obviously enriched in low-risk patients. Besides, the tumor purity was lower in low-risk patients (*P* < 0.001; Fig 6A). This result could be explained by a previous report showing samples with low tumor purity are related to high stromal and immune scores [23].

Subsequently, CIBERSORT and MCP-counter were used to explore the fraction of specific immune cell types in high- and low-risk patients. The comparisons between high- and low-risk patients were summarized in Fig 6B and 6C. The results showed that low-risk patients were detected having higher cell composition of CD8^+^ T cells in both methods (*P* < 0.0001). Besides, the fraction of activated memory CD4^+^ T cells (*P* < 0.01), T cells follicular helper (*P* < 0.0001), Cytotoxic lymphocytes (*P* < 0.001), B lineage (*P* < 0.001) and Myeloid dendritic cells (*P* < 0.01) were found having higher fraction in low-risk patients, whereas high-risk patients were more associated with macrophages M0 (*P* < 0.01) and activated mast cells (*P* < 0.05). The above results confirmed that ccHPS was related to the microenvironment remodeling in the tumor immune system in cervical cancer.

### Chemotherapy drug sensitivity between high- and low-risk groups

Tissue hypoxia is an indicator of adverse prognosis in cancer patients. Moreover, hypoxia could enhance the resistance to chemotherapy through a series of signaling biological processes, such as DNA damage, apoptosis, p53, autophagy and mitochondrial activity [7, 44, 45]. In this study, we compared the chemotherapy drug sensitivity in high- and low-risk groups using six chemotherapy drugs in cervical cancer, including Paclitaxel, Gemcitabine, Cisplatin, Gefitinib, Mitomycin C, and Sunitinib. Consistently, we observed significantly higher estimated IC50 levels in ccHPS high score patients for most chemotherapy drugs, including Paclitaxel, Gefitinib, Sunitinib, and Mitomycin C (S3 Fig), suggesting patients in high-risk groups are more resistant to these chemotherapy drugs. Meanwhile, we observed no significant difference in the estimated IC50 between the high and low ccHPS score groups for Cisplatin and Gemcitabine. We concluded that the low-risk patients were more sensitive to chemotherapy drugs and could benefit from Paclitaxel, Gefitinib, Sunitinib, and Mitomycin C therapy.

### Independent prognostic analysis

Univariate and multivariate Cox regression analyses were separately performed using TCGA-CESC entire cohort to investigate whether the risk score from ccHPS is an independent prognostic factor of OS with cervical cancer. The results were shown in Table 1. In univariate Cox regression analysis, the risk score was significantly associated with patient prognosis (*P* = 1.02×10^−6^). Besides, Age, FIGO stage, Lymphovascular invasion (LVI), and T stages are considerably associated with prognoses as well with the threshold *P* < 0.05 (Table 1). These significant clinical factors were then included in multivariate cox regression analysis, and the ccHPS risk score remained associated with patient OS (*P* = 1.33×10^−3^). The above findings suggested that ccHPS was an independent prognostic factor for patients with cervical cancer. Interestingly, LVI is an independent prognostic factor as well (Table 1).

**Table 1:** Results of the univariate and multivariate cox regression analyses of OS in the TCGA-CESC cohort.

Variables	Univariate cox analysis			Multivariate cox analysis		
	Coefficient	Hazard Ratio (95% CI)	*P*-value	Coefficient	Hazard Ratio (95% CI)	*P*-value
**ccHPS risk score** (Low)	**-1.31**	**0.27 (0.16–0.45)**	**1.02×10** ^**−6**^	**-1.49**	**0.23 (0.09–0.56)**	**1.33×10** ^**−3**^
**LVI** (Present)	**2.32**	**10.21 (2.41–43.30)**	**1.61×10** ^**−2**^	**2.39**	**10.93 (2.54–47.00)**	**1.31×10** ^**−3**^
**FIGO stage** (Stage II-IV)	**0.79**	**2.19 (1.33–3.61)**	**2.00×10** ^**−3**^	-0.34	0.71 (0.20–2.52)	0.59
**T stage** (T2-4)	**0.59**	**1.81 (1.03–3.18)**	**0.04**	-0.06	0.94 (0.33–2.71)	0.92
**Age** (>60)	**0.57**	**1.77 (1.05–2.97)**	**0.03**	0.93	2.55(0.82–7.86)	0.10
**Grade** (G3-4)	-0.11	0.89 (0.53–1.51)	0.68	-	-	-
**Radiation therapy** (YES)	-0.04	0.96 (0.45–2.07)	0.92	-	-	-

### Establishment and evaluation of the nomogram

**Fig 7:**
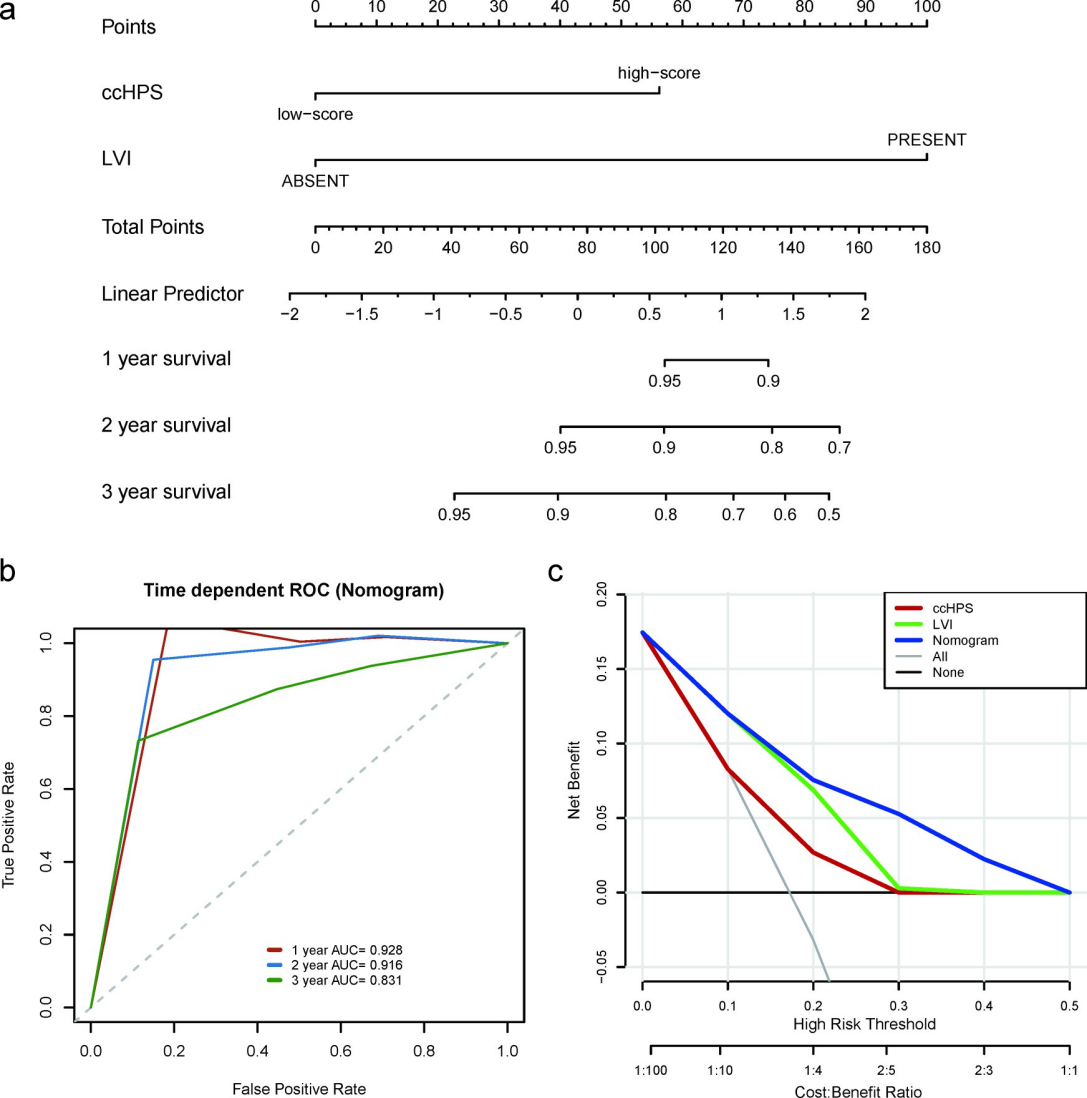
Development and evaluation of the nomogram with ccHPS and LVI using TCGA-CESC entire cohort. (a) The nomogram plot is based on ccHPS risk score and Lymphovascular invasion (LVI) for predicting the 1-, 2- and 3-years OS. (b) The time-dependent ROC curve shows the assessment of the nomogram in 1-, 2-, and 3-years OS. (c) Decision curve analysis separately for the nomogram, LVI, and ccHPS.

The results from Table 1 revealed that ccHPS and LVI are independent prognostic factors in cervical cancer. Accordingly, a nomogram integrating the ccHPS risk score and LVI was developed to predict the probability of 1-, 2- and 3-years overall survival in cervical cancer using TCGA entire cohort (Fig 7A). The nomogram was predicted well, with the C-index reach 0.835. Meanwhile, the ROC curve confirmed that the nomogram has a good performance in predicting OS with AUC at 1-, 2- and 3-years separately reach 0.928, 0.916 and 0.831 (Fig 7B). Besides, the DCA result in Fig 7C demonstrated that the prediction performance of the nomogram is better than that in one of the LVI and ccHPS risk scores.

## Discussion

Cervical cancer is a major public health problem worldwide among females, ranking fourth in incidence and death rates [1, 2]. Hypoxia is a typical microenvironment character in tumors with deprived adequate oxygen supply, and was shown to be a prognostic factor associated with adverse outcomes in cervical cancer [11–13]. Moreover, the expression changes of hypoxia-related genes were shown to be associated with cell invasion and metastasis in tumors [14, 15, 46]. Therefore, integrating hypoxia-related genes to investigate their effects on patient survival and the related biological changes might help us comprehensively understand the hypoxic tumor microenvironment in cervical cancer, giving us insights into improving cancer therapy. In this study, we constructed a hypoxia-based risk model including nine genes (EFNA1, IER3, ISG20, KLF7, LDHC, P4HA2, PGM1, RBPJ and STC1), which is developed and confirmed separately by the TCGA training cohort (n = 203) and two validation cohorts (TCGA-test cohort (n = 86) and CGCI-HTMCP-CC cohort (n = 117)) based on LASSO Cox regression analysis. The PCA analysis, ROC analysis, and K-M survival analysis suggested an excellent performance of the ccHPS model in predicting the prognosis of patients with cervical cancer. The high-risk score was considerably associated with adverse outcomes of the patients. The stable performance of the ccHPS model across different clinical factors further confirmed the robustness of its prognostic value. Importantly, univariate and multivariate Cox regression analysis showed that ccHPS was an independent prognostic factor in cervical cancer. Moreover, a nomogram integrating ccHPS and LVI was developed, which further improved the prediction performance, predicting the 1-, 2- and 3-year prognosis with AUC values of 0.928, 0.916 and 0.831, respectively. These findings demonstrated the high prognostic value of the hypoxia-related gene-based risk model. In addition to the prognostic value of the ccHPS model, ccHPS could also indicate tumor hypoxic microenvironment. The ccHPS score of both tumor samples and cell lines in a high hypoxic environment was consistently higher than those in a low hypoxic environment.

Furthermore, most of the nine genes in ccHPS have been reported to be associated with the tumor prognosis in different human cancer types, such as IER3 is a prognostic factor in bladder cancer [47], expression increased KLF7 promotes tumor cell growth and metastasis in pancreatic cancer [48] and tumor cell migration in oral squamous cell carcinoma [49], and STC1 is a prognostic predictor in patients with esophageal squamous cell carcinoma [50] and colorectal cancer [51]. Notably, P4HA2 has been reported to be a prognostic biomarker in cervical cancer, which was increased expression in cervical cancer tissues compared with the adjacent normal tissues, and related to poor prognosis [52, 53].

Tumor microenvironment (TME) is a complex biological environment where solid tumors are located, and increasing evidence showed that TME played critical roles in tumor progression [54]. As a dominant microenvironmental factor, hypoxia is considered the most relevant factor in tumor progression and metastasis [55, 56]. Consistently, of the ccHPS risk model with nine hypoxia-related genes, the high-risk score is remarkably related to the poor prognosis of cervical cancer patients, indicating the TME remodeling ability of hypoxia and a complicated biological change within TME that ccHPS might derive. An increasing number of studies indicated that hypoxia plays an essential role in tumor immune escape with the infiltration of large amounts of immunosuppressive cells in hypoxic zone of solid tumors, including tumor-associated macrophages and myeloid-derived suppressor cells [55–57]. Moreover, hypoxia reduces the proliferation and differentiation ability of CD8^+^ T cells [55, 58], a preferred antitumor immune cell, thus leading to adverse prognosis in cancer [59, 60]. Accordingly, we explored the correlation of ccHPS with immune systems in TME using ESTIMATE. The results showed that high-risk patients had lower immune scores and higher tumor purity than low-risk patients, suggesting a negative correlation between ccHPS risk score and antitumor immunity of cervical cancer. Further CIBERSORT and MCP-counter analysis showed that CD8^+^ T cells, activated memory CD4^+^ T cells, T cells follicular helper, Cytotoxic lymphocytes, B lineage, and Myeloid dendritic cells were having a higher fraction in low-risk patients with cervical cancer. In contrast, high-risk patients were more associated with the accumulation of macrophages M0 and activated mast cells. Previous reports have consistently shown that mast cells, an cancer immunotherapy target, can be activated by hypoxia in solid tumors [61]. Hypoxia could also lead to the accumulation of macrophages [7, 56, 62], and macrophages M0 promotes cancer invasion [63]. The results suggested the close connection between hypoxia and ccHPS with similar remodeled immunogenic features, including increased immunosuppressive cells and decreased antitumor immune cells. Therefore, we concluded that ccHPS might give us insights into antitumor immunotherapy and further improve the strategies for treating cervical cancer.

Besides the effects on the tumor immunity, the ccHPS model might be an indicator for remodeling TME with cancer cell metabolism. Previous reports showed that hypoxia induces proteomic changes in tumor cells, thus leading to a series of metabolic changes to stimulate tumor growth and enhance therapy resistance [64–66]. In this study, GSEA analysis between high-risk and low-risk patients divided by ccHPS confirmed the effects of ccHPS on remodeling TME with cancer cell metabolism. In the results, a series of pathways in tumor progression and metastasis were enriched in high-risk patients, such as EMT [36], keratinization [38, 39], cytokine-cytokine receptor interaction pathway [40] and ribosome [41–43]. Therefore, further investigation of the ccHPS-led metabolic changes in tumor cells will facilitate us to focus on specific pathways, and finally direct the tumor therapy.

In conclusion, the present study integrated hypoxia-related genes to construct a risk model, ccHPS, which was developed and confirmed separately by the training cohort and two independent test cohorts based on LASSO Cox regression analysis. Notably, the ccHPS model could be an independent prognosis factor in predicting patient OS with cervical cancer and closely related to the TME remodeling in cell metabolism and tumor immunity. We concluded that the ccHPS risk model might help us better understand the hypoxic tumor microenvironment, provide insights into antitumor immunotherapy, and ultimately improve the treatment strategies of cervical cancer.

## Supporting information

S1 Fig Principal component analysis of the gene expression profile in high-risk and low-risk patients. (a) PCA analysis for high-risk and low-risk patients in TCGA-Training cohort. (b) PCA in TCGA-test cohort. (c) PCA in TCGA-CESC entire cohort. The genes used for PCA analysis were the nine genes in the ccHPS model.
(TIF)

S2 Fig Kaplan-Meier curve analysis for the high-risk and low-risk patients stratified by clinical factors. The clinical factors included Grade in stages G1-2 (a) and G3-4 (b), Age that higher than 50 (c) and less than 50 years old (d), N stages in N0 (e) and N1 (f), T stages in T1 (g) and T2-4 (h). The high-risk and low-risk scores are divided by the median score in ccHPS using the TCGA-CESC cohort.
(TIF)

S3 Fig Box plots showed the differences between high- and low-ccHPS scores in the estimated IC50 levels of six chemotherapy drugs using TCGA-CESC patients. The statistical comparisons were conducted using the Wilcoxon test. **** *P* ≤ 0.0001, *** *P* ≤ 0.001, ** *P* ≤ 0.01 and * *P* ≤ 0.05. and ns *P* > 0.05.
(TIF)

S1 Table The training and validation datasets in TCGA-CESC and CGCI-HTMCP-CC. (XLSX)

S2 Table The gene identifier mapping table used for converting Ensembl gene identifier into gene symbol, which was based on Human GENCODE database (version 22). (XLSX)

S3 Table Clinical characteristics of patients in TCGA training and test cohorts, and CGCI-HTMCP-CC cohort. (DOCX)

S4 Table Univariate cox analysis result for 190 hypoxia-related genes covered in TCGA-CESC cohort. (XLSX)

S5 Table Gene Set Enrichment Analysis (GSEA) using the MSigDB gene sets derived from KEGG, HALLMARK, and gene ontology (biological process). (XLSX)
