# Supplementary figures and images for: Identification and validation of a prognostic signature related to hypoxic tumor microenvironment in cervical cancer
Source: PLoS One. 2022 Jun 3;17(6):e0269462. doi: 10.1371/journal.pone.0269462 (PMC9165826; doi:10.1371/journal.pone.0269462)

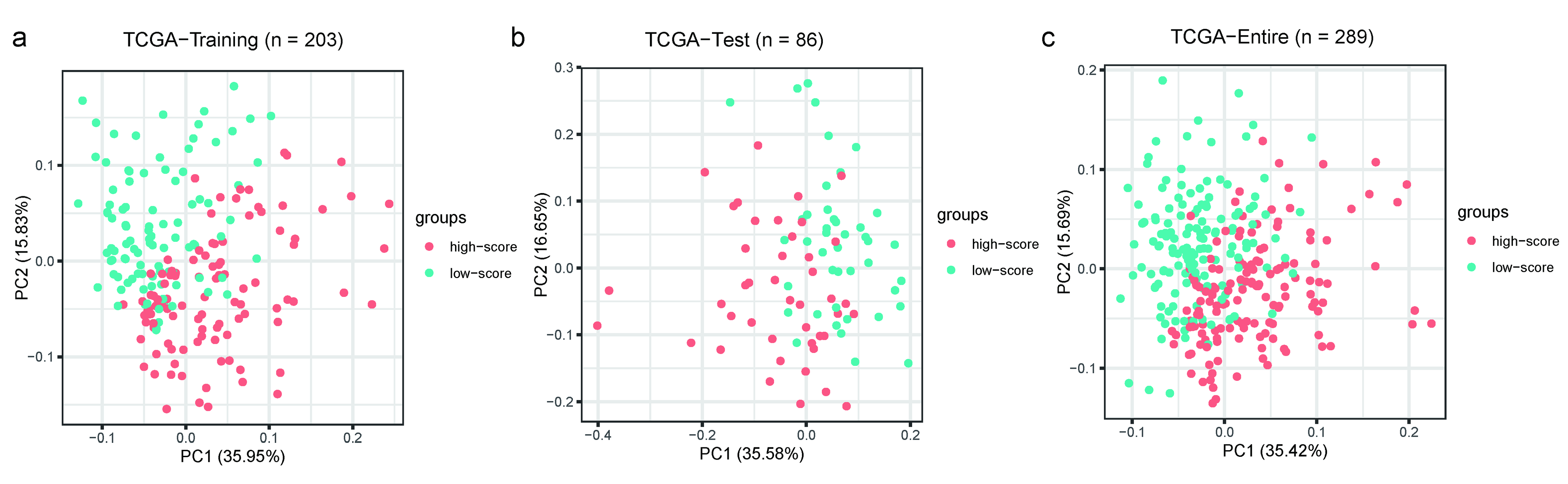

Supplement: S1 Fig — (a) PCA analysis for high-risk and low-risk patients in TCGA-Training cohort. (b) PCA in TCGA-test cohort. (c) PCA in TCGA-CESC entire cohort. The genes used for PCA analysis were the nine genes in the ccHPS model. (TIF) [file pone.0269462.s001.tif]

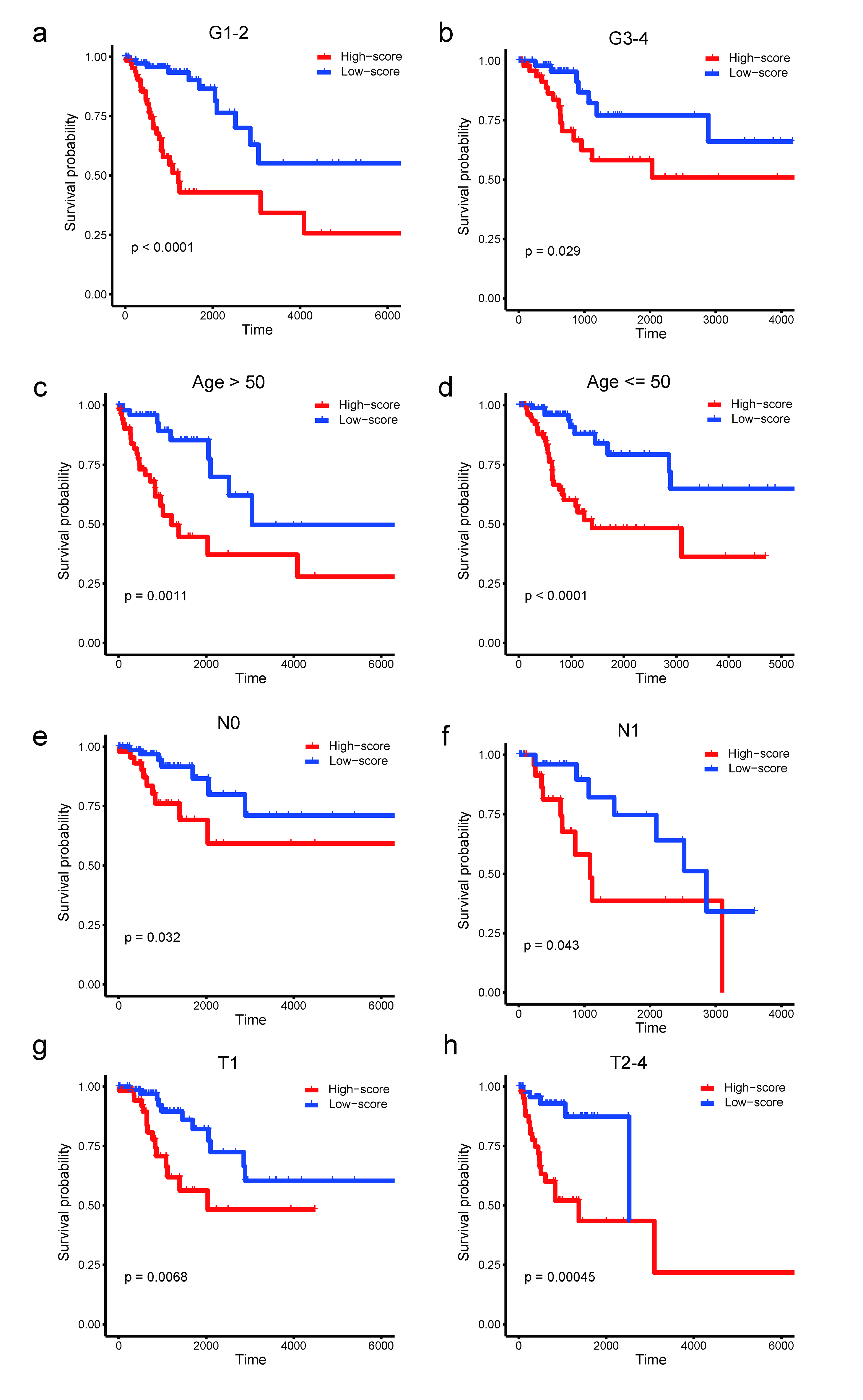

Supplement: S2 Fig — The clinical factors included Grade in stages G1-2 (a) and G3-4 (b), Age that higher than 50 (c) and less than 50 years old (d), N stages in N0 (e) and N1 (f), T stages in T1 (g) and T2-4 (h). The high-risk and low-risk scores are divided by the median score in ccHPS using the TCGA-CESC cohort. (TIF) [file pone.0269462.s002.tif]

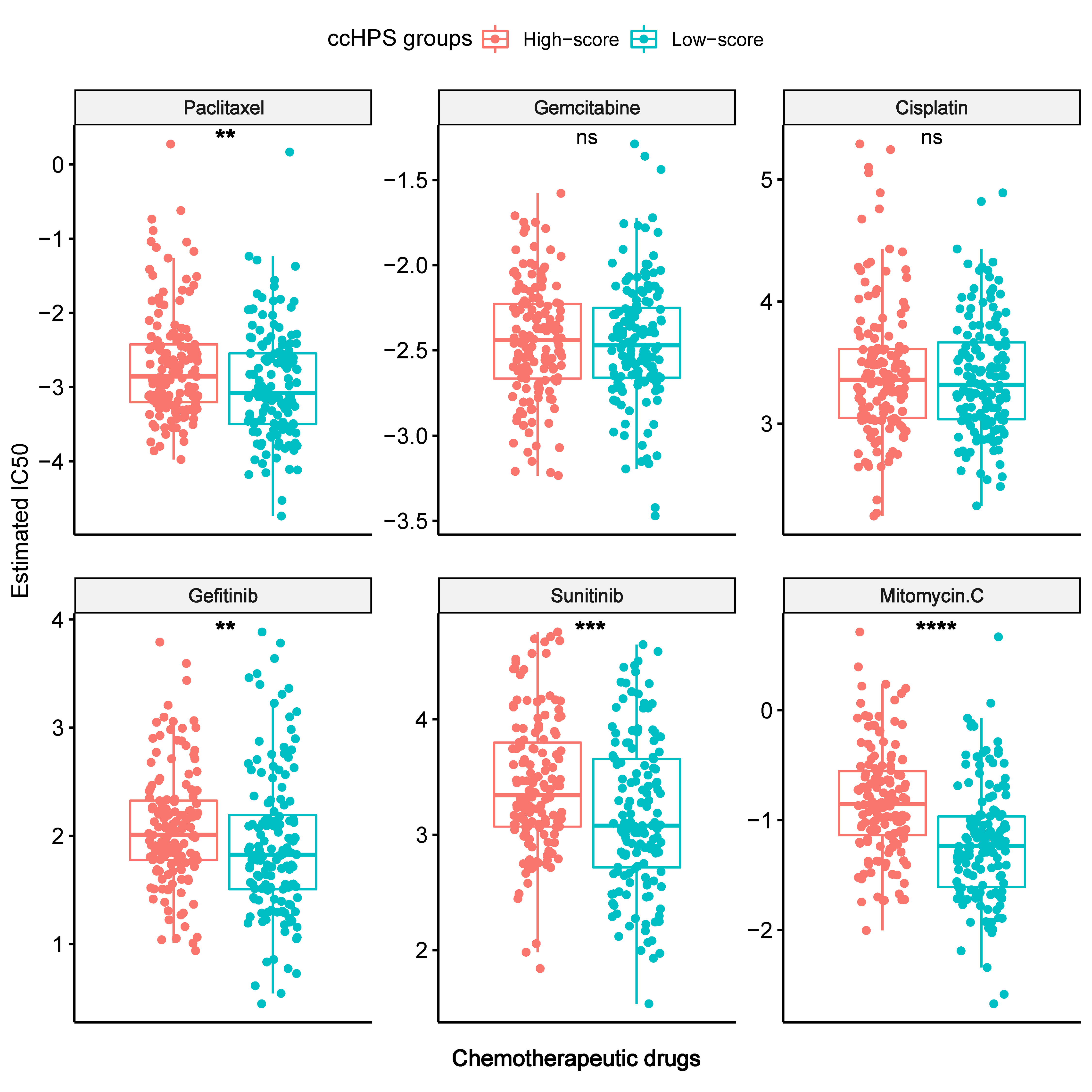

Supplement: S3 Fig — The statistical comparisons were conducted using the Wilcoxon test. **** P ≤ 0.0001, *** P ≤ 0.001, ** P ≤ 0.01 and * P ≤ 0.05. and ns P > 0.05. (TIF) [file pone.0269462.s003.tif]
