# Supplementary material for: Identification and validation of a prognostic signature related to hypoxic tumor microenvironment in cervical cancer
Source: PLoS One. 2022 Jun 3;17(6):e0269462. doi: 10.1371/journal.pone.0269462 (PMC9165826; doi:10.1371/journal.pone.0269462)
Supplement: S3 Table — (DOCX) [file pone.0269462.s006.docx]

**S3 Table. Clinical characteristics of patients in TCGA training and test cohorts, and CGCI-HTMCP-CC cohort.**

|  |  | **TCGA-CESC cohort** | |  | **CGCI-HTMCP-CC cohort** |
| --- | --- | --- | --- | --- | --- |
| **Variables** |  | **Training**  **No. (%)** | **Test**  **No. (%)** | **P-value** | **No. (%)** |
| **Number of patients** |  | 203 | 86 |  | 117 |
| **Age** | **<= 50** | 126 (62.07) | 54 (62.79) | 1.00 | 79 (67.52) |
|  | **> 50** | 77 (37.97) | 32 (37.21) |  | 37 (31.62) |
| **OS** | **Alive** | 151 (74.38) | 67 (77.91) | 0.63 | 44 (37.61) |
|  | **Dead** | 52 (25.62) | 19 (22.09) |  | 73 (62.39) |
| **FIGO stage** | **Stage I-II** | 155 (76.35) | 67 (77.91) | 1.00 | 72 (61.54) |
|  | **Stage III-IV** | 43 (21.18) | 18 (20.93) |  | 45 (38.46) |
| **M stage** | **M0** | 74 (36.45) | 33 (38.37) | 1.00 | 27 (23.08) |
|  | **M1** | 7 (3.45) | 3 (3.49) |  | 2 (1.71) |
| **T stage** | **T1** | 87 (42.86) | 50 (58.14) | 0.06 | 18 (15.38) |
|  | **T2-4** | 71 (34.98) | 22 (25.58) |  | 99 (84.62) |
| **N stage** | **N0** | 86 (42.36) | 42 (48.84) | 0.95 | 21 (17.95) |
|  | **N1** | 36 (17.73) | 19 (22.09) |  | 11 (9.40) |
| **Grade** | **G1-2** | 99 (48.77) | 46 (53.49) | 0.43 | 62 (52.99) |
|  | **G3-4** | 86 (42.36) | 31 (36.05) |  | 51 (43.59) |
| **Lymphovascular invasion** | **Present** | 54 (26.60) | 25 (29.07) | 0.73 | - |
|  | **Absent** | 45 (22.17) | 25 (29.07) |  | - |
| **Radiation therapy** | **YES** | 102 (50.25) | 40 (46.51) | 0.18 | - |
|  | **NO** | 34 (16.75) | 22 (25.58) |  | - |
